# *let-7e* downregulation characterizes early phase colonic adenoma in APC^Min/+^ mice and human FAP subjects

**DOI:** 10.1371/journal.pone.0249238

**Published:** 2021-04-26

**Authors:** Annalisa Contursi, Maria Arconzo, Marica Cariello, Marilidia Piglionica, Simona D’Amore, Michele Vacca, Giusi Graziano, Raffaella Maria Gadaleta, Rosa Valanzano, Renato Mariani-Costantini, Gaetano Villani, Antonio Moschetta, Elena Piccinin

**Affiliations:** 1 Department of Interdisciplinary Medicine, University of Bari “Aldo Moro”, Bari, Italy; 2 Fondazione Mario Negri Sud, Santa Maria Imbaro (CH), Italy; 3 INBB, National Institute for Biostructures and Biosystems, Rome, Italy; 4 Department of Biomedical Sciences and Human Oncology (DIMO), Medical Genetics, University of Bari "Aldo Moro", Bari, Italy; 5 Department of Clinical Physiopathology, University of Florence, Florence, Italy; 6 Center for Advanced Studies and Technology (C.A.S.T.), University “G. d’Annunzio”, Chieti-Pescara, Chieti, Italy; 7 Department of Basic Medical Sciences, Neurosciences and Sense Organs, University of Bari "Aldo Moro", Bari, Italy; Université Clermont Auvergne - Faculté de Biologie, FRANCE

## Abstract

The crypt-villus axis represents the essential unit of the small intestine, which integrity and functions are fundamental to assure tissue and whole-body homeostasis. Disruption of pathways regulating the fine balance between proliferation and differentiation results in diseases development. Nowadays, it is well established that microRNAs (miRNAs) play a crucial role in the homeostasis maintenance and perturbation of their levels may promote tumor development. Here, by using microarray technology, we analysed the miRNAs differentially expressed between the crypt and the villus in mice ileum. The emerged miRNAs were further validated by Real Time qPCR in mouse model (Apc^Min/+^), human cell lines and human tissue samples (FAP) of colorectal cancer (CRC). Our results indicated that miRNAs more expressed in the villi compartment are negatively regulated in tumor specimens, thus suggesting a close association between these microRNAs and the differentiation process. Particularly, from our analysis *let-7e* appeared to be a promising target for possible future therapies and a valuable marker for tumor staging, being upregulated in differentiated cells and downregulated in early-stage colonic adenoma samples.

## 1. Introduction

The epithelium of small intestine is organized into simple repetition of crypt-villus units. This architecture is designed to maximize the absorptive capacity, with digitiform protrusions (villi) surrounded by epithelial invaginations (crypt of Lieberkühn), where proliferative stem cells reside. Stem cells guarantee the rapid self-renewal of the entire intestinal epithelium, thus promoting the maintenance of the intestinal barrier as well as absorptive and defensive functions [1]. Within 5 days, stem cells migrate from the bottom of the crypt along the villus in a dynamic process of progressive differentiation. Once reached the top of the villi, cells undergo apoptosis, and are shed into the intestinal lumen [1,2]. The fine interplay between proliferation and differentiation is under the control of several important pathways, such as Wnt, Notch and Hedgehog signalling pathways [3–6]. The perturbation of the fine balance between proliferation and differentiation can lead to hyperproliferation and ultimately tumor growth.

Since today, different factors that control cell fate and development have been identified [7,8]. Among them, microRNAs have emerged as post-transcriptional regulators of gene expression, which balance is fundamental to maintain intestinal homeostasis.

MicroRNAs (miRNAs) are 19–25 nucleotides long single-stranded non coding RNA molecules, which function is to modulate target mRNA translation and stability [9]. Since their discovery, it has emerged that more than one-third of the human genes are regulated by miRNAs [10]. Indeed, they control a large plethora of physiological processes aimed to maintain organism homeostasis, and involving development, differentiation, metabolism, as well as cell cycle growth and death [11]. MicroRNAs expression is tissue specific and developmentally regulated, thus alteration of their levels is closely associated to diseases, including cancer [12–14]. The rapid advancement of high-throughput techniques has facilitated the identification of microRNAs whose expression is altered in several type of cancers or different between primary tumor and metastasis [15–19].

In the gut, microRNAs expression is essential for maintaining intestinal homeostasis. Indeed, in mice the intestinal ablation of *Dicer 1*, which catalyses a committed step in miRNA biosynthesis, caused a compromised integrity of the intestinal barrier, resulting in inflammation with lymphocytes and neutrophils infiltration [20]. Moreover, intestinal H^+^-coupled oligo-peptide transporter (*PepT1*)-knockout mice displayed an altered distribution of miRNAs along the crypt-villus axis, with consequent changes in the miRNA profiles of both villi and crypts. This results in shorten intestinal microvilli and reduced body weight [21].

Here, by using a miRNAs microarray approach, we reported miRNAs differentially regulated along the crypt-villus axis in mice. Candidate miRNAs showing human sequence homology were subsequently validated in the colon carcinoma HT29 and Caco2 cell line. Then, we assessed the expression of miRNAs in Apc^Min/+^ mice, a mouse model of spontaneous colon cancer, as well as in FAP patients (tumor vs. normal mucosa). Overall, our data highlighted novel candidate biomarkers of enterocyte differentiation and proliferation, together with putative targets responsible for early-stage adenomas.

## 2. Materials and methods

### 2.1 Animals

All the experiments presented in this study were carried out in C57BL/6J mice and Apc^Min/+^ male mice (Jackson Laboratory). For crypt-villus experiments, mice were sacrificed at 4 months of age. For CRC experiments, mice were sacrificed at 6 months of age. All mice were housed with a standard diet provided *ad libitum* and examined daily. The Ethical Committee of the University of Bari approved this experimental set-up, which also was certified by the Italian Ministry of Health in accordance with internationally accepted guidelines for animal care.

### 2.2 Isolation of the murine epithelium from the crypt to villus region

After sacrifice, the ileum was removed from each mouse, flushed with cold saline solution, and longitudinally cut. The epithelium from the crypt-villus axis was isolated following a modified Weiser method. Briefly, ileum was cut longitudinally and transferred into Hank’s balanced salt solution with 0.5mM-DTT to remove the luminal content (mucus and faeces). Then, ileum was transferred into 30 ml of Chelating Buffer (Na2PO4 5mM, NaCl 96mm, Na Citrate 27mM, KH2PO4 8mM, KCl 1.5 mm, D-Sorbitol 55mM, Sucrose 44mM, DTT 0.5 mM), and incubated at 4°C with constant stirring for 20 minutes. Supernatant was collected and called “V0 fraction”. Intestinal fragment was transferred into a falcon (50 ml) with 15 ml of Chelating buffer and gently shaken by hand (30 inversions). Supernatant was collected twice for each fraction (V1, V2, V3 and C1). All collected fractions were centrifuged at 1000g and the resultant pellets were immediately processed for RNA extraction.

### 2.3 Familial adenomatous polyposis patients

We received tissue samples of Familial Adenomatous Polyposis (FAP) patients (tumours and normal intestinal mucosa) from Dr. R. Valanzano (University of Florence, Florence, Italy) and Dr. R. Mariani-Costantini (University of Chieti-Pescara, Chieti, Italy). Unrelated Italian patients with FAP were recruited for the study after approval by the Ethical Committee of the University “G. D’Annunzio” of Chieti. Written informed consent was obtained from each patient. All the patients presented with a clinical diagnosis of FAP with histo-morphologic confirmation of colonic adenomas and with a characterized germline mutation in the APC gene.

### 2.4 Cell line and differentiation

The colon carcinoma HT29 (ATCC® HTB-38™) and Caco2 (ATCC® HTB-37™) cell lines were both purchased from American Type Culture Collection. HT29 cells were maintained in Dulbecco’s modified Eagle’s medium (DMEM, Thermo Fisher Scientific, Massachusetts, USA) supplemented with 10% fetal bovine serum (FBS, Thermo Fisher Scientific, Massachusetts, USA) and 1% Penicillin-Streptomycin (Thermo Fisher Scientific, Massachusetts, USA). Caco2 cells were maintained in Minimum Essential Medium Eagle (MEM, Sigma-Aldrich, Missouri, USA) supplemented with 20% FBS, 1% Penicillin-Streptomicin and L-Glutamine. HT29 and Caco2 differentiation was achieved by spontaneous post-confluence growth arrest. Briefly, 5x10^4^ cells *per well* were seeded in a 6 multi-well plate. Cells were harvested at day 3 (pre-confluence), day 7 (confluence), day 14 (post-confluence) after seeding and processed for RNA extraction. Each single experiment was conducted in triplicate. Results are expressed as mean of 3 independent experiments.

### 2.5 RNA extraction

Total RNA was isolated by Qiazol reagent (Qiagen, Hilden, Germany) following the manufacture’s instruction. To avoid possible DNA contaminations, RNA was treated with DNAase I (Thermo Fisher Scientific, Massachusetts, USA). RNA purity and concentration were checked by spectrophotometer, while the RNA integrity was assessed by Bio-RadExperion™ (Bio-Rad, Hercules, CA). Only samples with Relative Quality Index (RQI) N8 were used for microarray analysis. Samples were stored in aliquot at −80°C prior to use.

### 2.6 Microarray analysis of miRNA expression profile

Microarray miRNA expression analysis was performed on RNA extracted from the different fractions of ileum of wild type mice. Whole RNA (400 ng) was studied using the Illumina Universal MicroRNA Expression Profiling. The bead chips were read on the Illumina iScan System microarray platform (Illumina, San Diego, CA, USA). Upon the manufacturer instructions, data were processed using the Illumina Genome Studio Software through specific algorithms of filtration and cleaning of the signal. All the items with detection p-value >0.01 were excluded. Data were normalized together with the quantile method. Background was not subtracted. Final output consisted of normalized fluorescence intensity of each probe (AVG signal), representing the expression levels of each miRNA. The data discussed in this publication have been deposited in NCBI’s Gene Expression Omnibus and are accessible through GEO Series accession number GSE165991 (https://www.ncbi.nlm.nih.gov/geo/query/acc.cgi?acc=GSE165991).

### 2.7 Quantitative real-time Polymerase Chain Reaction (RTqPCR) for miRNAs

For miRNA expression analysis, reverse transcription of 10 ng of RNA was performed using TaqMan microRNA Reverse Transcription Kit (Thermo Fisher Scientific, Massachusetts, USA), following the manufacturer instructions. Real Time qPCR assays were performed in 96-well optical reaction plates using a Quantum5 machine (Thermo Fisher Scientific, Massachusetts, USA). qPCR assays were conducted in triplicate wells for each sample, using pre-validated TaqMan Assays and TaqMan Universal Master Mix (Thermo Fisher Scientific, Massachusetts, USA), according to the manufacturer instructions. sno202, U6snRNA and RNU44 were used as internal controls. Relative quantification was carried out using the ΔΔCT method. For mRNA expression analysis, complementary DNA (cDNA) was synthesized by retro-transcribing total RNA using the High-Capacity DNA Kit (Thermo Fisher Scientific), following the manufacturer’s instruction. qPCR assays were conducted in triplicate wells for each sample, using validated primers (Table 1) and intercalated dye (Power Sybr Green, Thermo Fisher Scientific). Cyclophilin A was used as an internal control for mouse studies, whereas Cyclophilin B was used as an internal control for human studies. Relative quantification was performed using the ΔΔCT method.

**Table 1:** List of validated primers used.

Gene	FW	RV	Organism
Cyclophilin B	GGCCAACGATAAGAAGAAGGG	ACAAAATTATCCACTGTTTTTGGAACA	Mus Musculus
Cyclin D1	CATCCATGCGGAAAATCGT	TCTACGCACTTCTGCTCCTCA	Mus Musculus
c-Myc	TGTATGTGGAGCGGTTTCTCA	CTGGTAGGAGGCCAGCTTCT	Mus Musculus
I-Fabp	GGTGACAACTTTCAAAGGCATAAA	TGTCGCCCAATGTCATGGTA	Mus Musculus
Klf4	CAGACCAGATGCAGTCACAAGTC	TGGGCTCCTCTGGCAGG	Mus Musculus
p21	GAACATCTCAGGGCCGAAAA	CAATCTGCGCTTGGAGTGATAG	Mus Musculus
Cyclophilin A	TTTCATCTGCACTGCCAAGA	TTGCAAAACACCACATGCT	Homo Sapiens
Cyclin D1	CGTGGCCTCTAAGATGAAGGA	CGGTGTAGATGCACAGCTTCT	Homo Sapiens
c-Myc	CCACCACCAGCAGCGACT	CAGAAACAACATCGATTTCTTCCTC	Homo Sapiens
I-Fabp	CGGAAATCGTGCAGAATGG	TTTGGACCCAGCGGTGAT	Homo Sapiens
Klf4	AGATGCAGCCGCAAGTCC	TCCTCTGGCATGCAGGAAC	Homo Sapiens
p21	GAAAACGGCGGCAGACC	CAGCCGGCGTTTGGAGT	Homo Sapiens

### 2.8 Datasets

In the present study, publicly available microRNA expression profiles were obtained from Gene Expression Omnibus (GEO Accession Number: GSE73487; GSE83924; GSE115513), where fresh frozen or FFPE colonic biopsy tissues (normal mucosa, adenoma and colorectal carcinoma) were collected from patients undergoing colonoscopy.

### 2.9 Statistical analysis

Microarray data were processed through specific algorithms of filtration and cleaning of the signal of the Illumina Genome Studio Software. Final output consisted of fluorescence intensity of each probe (AVG signal), representing the expression levels of each gene. “Differential Expression Analysis” with the “Illumina-custom error model” and with/without False Discovery Rate to adjust the p-value was used to select all the genes differentially expressed between the different intestinal fractions.

RTqPCR results are expressed as mean ± SEM as indicated in the figure legends. Statistical significance was determined by the paired Mann Whitney test or ANOVA analysis of variance (Kruskal-Wallis) with Dunn’s post hoc test. p-values (p<0.05, p<0.01, p<0.001) are considered significant. All statistical calculations were performed with GraphPad 5.00 for Windows software (GraphPad Software, San Diego California USA).

## 3. Results

### 3.1 Differential expression of microRNA in the crypt-villus axis in mice

**Fig 1:**
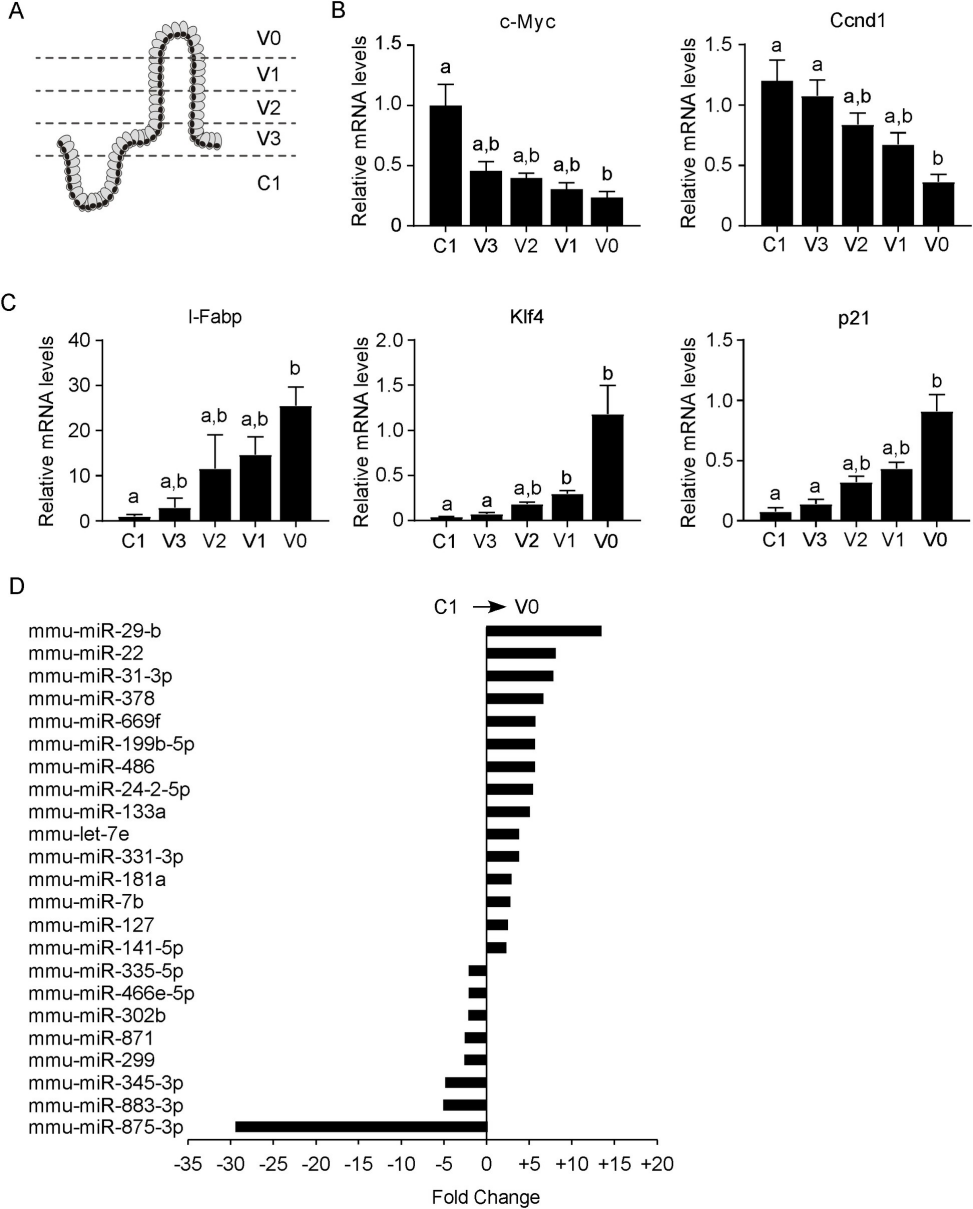
Identification of microRNAs differentially expressed along the crypt-villus axis in mice. Ileum from four months aged C57BL/6J wild type mice was processed to isolate epithelium from the crypt to villus region, and subsequently analysed for microarray microRNAs expression. (A) Schematic representation of fractioning of intestinal epithelium. (B) To validate the fractioning of the intestinal epithelium, we measured by Real Time qPCR the expression of Intestinal fatty-acid binding protein *(I-Fabp)*, Cyclin D1 *(Ccnd1)*¸ *c-Myc*, Kruppel-like factor 4 *(Klf4)*, and *p21*. *I-Fabp* and *Ccnd1* are markers of enterocyte differentiation in the apical compartment, while *c-Myc*, *Klf4* and *p21* were used as a marker of cell proliferation in the undifferentiated compartment. *Cyclophilin B* was used as a housekeeping gene to normalize data. Comparison of different fractions of wild-type (n = 6) was performed using Kruskal Wallis test followed by Dunn’s post-test. Results are expressed as mean ± SEM. Data from groups sharing the same lowercase letters were not significantly different, whereas data from groups with different case letters were significantly different (p<0.05). (C) miRNA expression chart along the crypt-villus axis, comparing the purified crypt fraction (C1) to the fully differentiated compartment of the villus (V0). The y-axis contains the name of the microRNA while the x-axis represents the fold change in expression. All microRNA identified were significantly changed.

To characterize the miRNAs signature along the crypt-villus axis, we firstly isolated the different fractions of the intestinal epithelium, from the V0 fraction, corresponding to the fully differentiated enterocytes at the top of the villus, to the C1 fraction, equivalent to the enterocytes in the proliferative crypt compartment (Fig 1A). Then, we validated the fractions obtained by studying the expression levels of well-studied enterocytes differentiation and proliferation markers, namely Intestinal-type fatty acid binding protein *(I-Fabp)*, Cyclin D1 *(Ccnd1)¸ c-Myc*, Kruppel-like factor 4 *(Klf4)*, and *p21*. As expected, the expression of the differentiation markers *I-Fabp* and *Ccnd1* increased moving along the crypt-villus axis, displaying the highest levels in V0 fraction. On the contrary, the proliferation markers *c-Myc*, *Klf4* and *p21* showed the opposite expression pattern, displaying the highest expression in C1 fraction (Fig 1B). Thus, our results are in line with previous data reporting a progressive differentiation of enterocytes along the crypt-villus axis.

Using the Illumina microarrays, we generated a miRNA expression chart along the crypt-villus axis, comparing the purified crypt fraction (C1) to the fully differentiated compartment of the villus (V0). We performed a non-parametric paired comparison on log2 normalized data, with significance adjusted for multiple comparisons (FDR), and we considered significant only those genes with fold changes ≤2 and an FDR ≤0.05. This analysis allowed us to identify a panel of differentially expressed miRNAs along the crypt-villus axis; in particular, we found 15 upregulated miRNAs characterizing the different cellular fractions, and 8 downregulated miRNAs throughout cell migration along the crypt-villus axis (Fig 1C).

**Fig 2:**
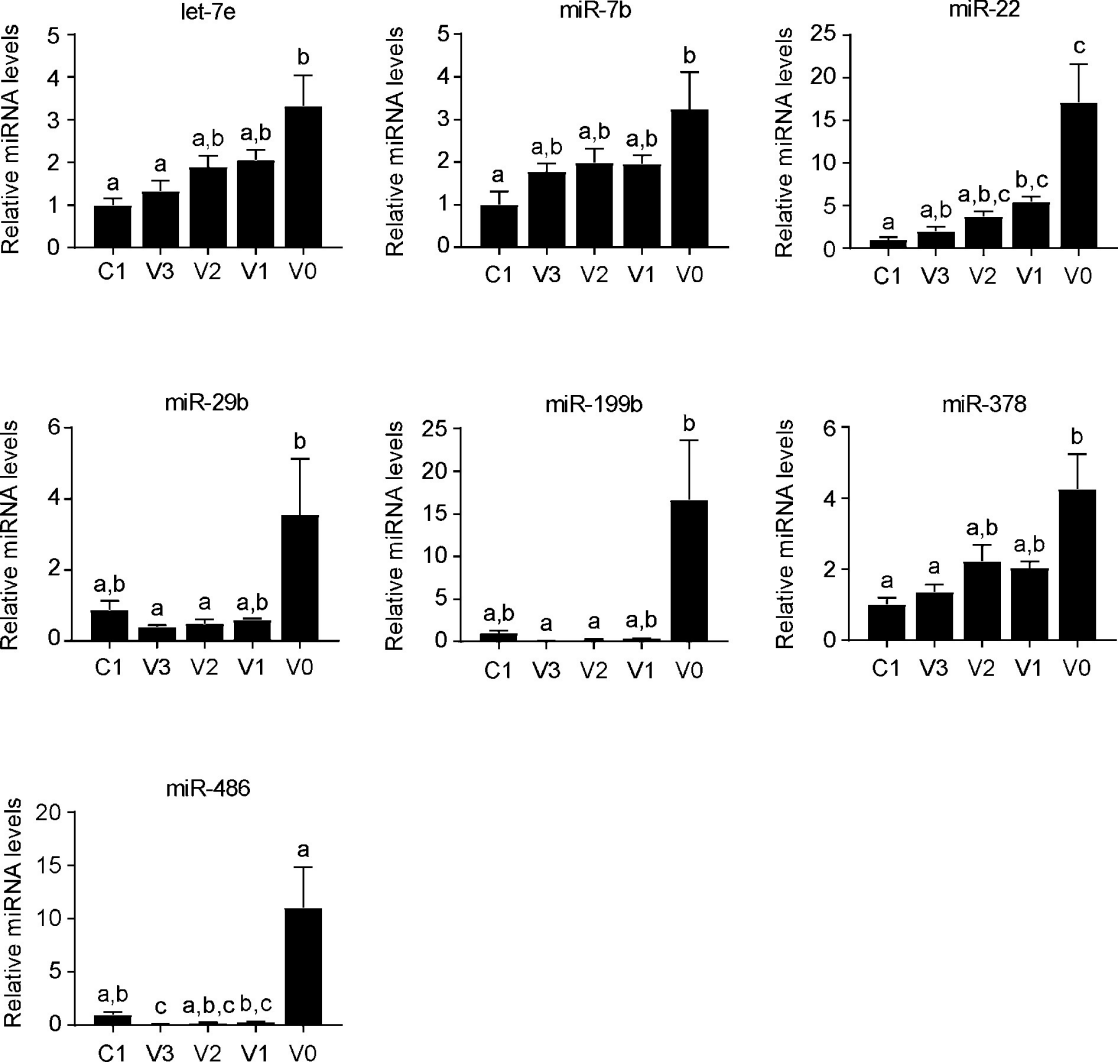
Validation of microRNA differentially expressed along the crypt-villus axis in mice. Ileum from four months aged C57BL/6J wild type mice was processed to isolate epithelium from the crypt to villus region. RNA extracted from each fraction was tested by Real Time qPCR to assess the expression levels of microRNAs found upregulated in microarray analysis. Quantitative normalization was performed using *sno202* as internal control. Comparison of different fractions of wild-type (n = 6) was performed using Kruskal Wallis test followed by Dunn’s post-test. Results are expressed as mean ± SEM. Data from groups sharing the same lowercase letters were not significantly different, whereas data from groups with different case letters were significantly different (p<0.05).

To investigate functional relationships in the set of differentially expressed miRNAs, we used the Ingenuity Pathway Analysis Software interrogating the Target Scan database. This tool is provided by Massachusetts Institute of Technology (MIT) for the *in-silico* prediction of miRNA targets and to study the involvement of miRNAs in pathways and networks. This bioinformatics analysis allowed us to establish the implication of differentially expressed miRNAs within a particular pathway. In particular, miRNA up-regulated in the villus were clustered in networks involved in cellular differentiation and development, while miRNAs suppressed in the villus were poorly characterized in terms of known target genes and involvement in specific pathways. This integrated approach allowed us to point to 7 miRNAs upregulated in the differentiated compartment (*miR-7b*, *let-7e*, *miR-486*, *miR-199b-5p*, *miR-378*, *miR-22*, *miR-29b*) that have been involved in cell differentiation, metabolism and/or cancer suppression, and 2 miRNAs (*miR-302b and miR-335-5p*) suppressed in the villus that have been highlighted as inhibitors of apoptosis or cancer development. The differential expression of 7 up-regulated miRNAs (l*et-7e*, *miR-7b*, *miR-22*, *miR-29b*, *miR-199b-5p*, *miR-378* and *mir-486*) in the crypt-villus axis was validated on the different cell fractions using TaqMan probes. All the miRNAs resulting up-regulated in the differentiated intestinal fractions in the microarray dataset showed an increasing gradient of expression moving from the crypt to the villus, where they display their highest levels (Fig 2). On the other hand, we were not able to validate the 2 down-regulated miRNAs of the microarray dataset (*miR-335-5p* and *miR-302b*), probably due to their low expression levels in the intestinal fractions.

### 3.2 Expression of microRNAs in HT29 and Caco2 during confluence-induced growth arrest

**Fig 3:**
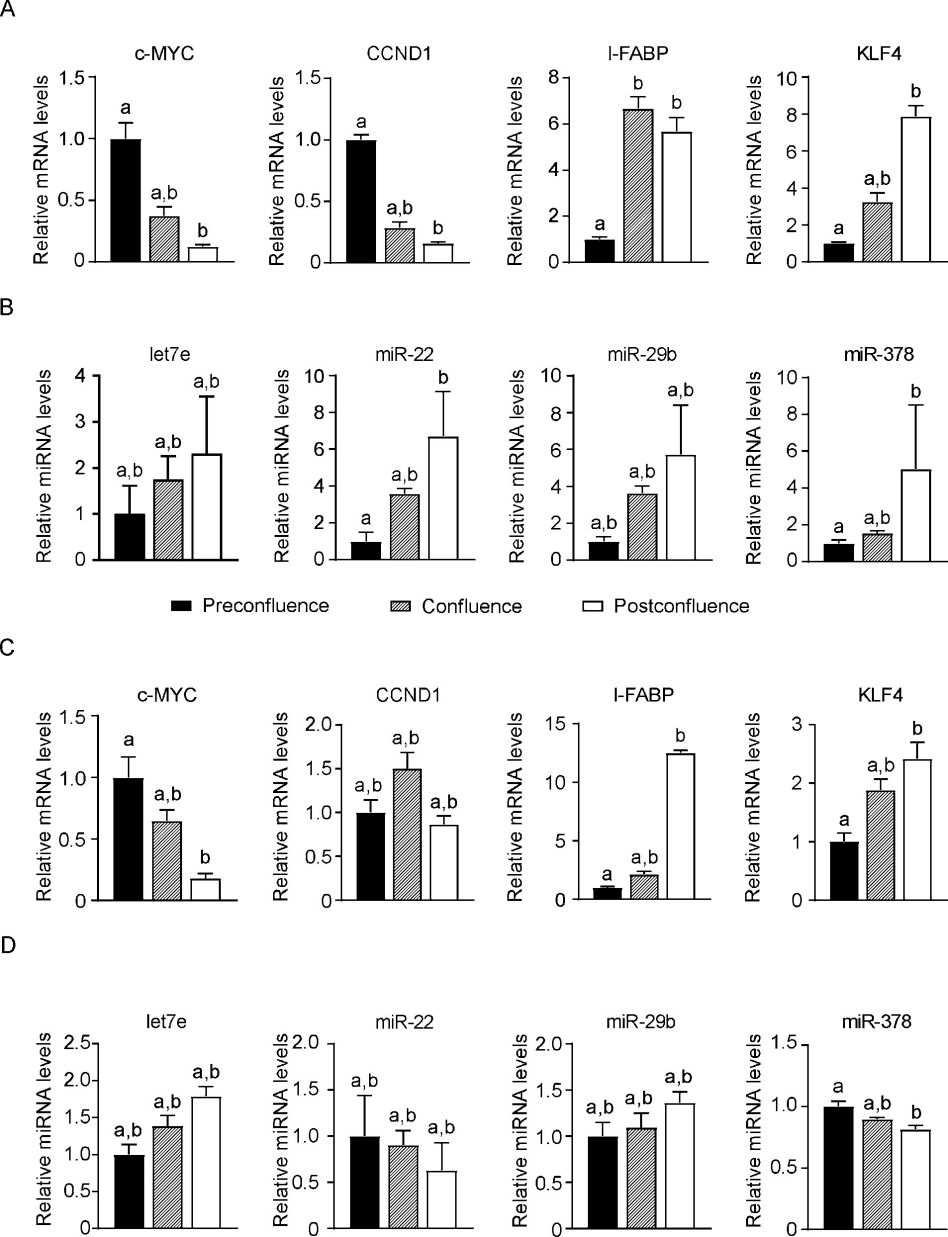
Expression of microRNAs in HT29 and Caco2 colon cancer cell lines during confluence-induced growth arrest. Real Time qPCR analysis of gene and microRNA differentially expressed in human HT29 and Caco2 colon cancer cell lines in pre-confluence, confluence and post-confluence phases. (A) Expression levels of proliferation markers *c-MYC* and cyclin D1 (*CCND1*) together with differentiation markers Intestinal fatty-acid binding protein (*I-FABP)* and Kruppel-like factor 4 (*KLF4*) in the different confluence phases of HT29. *Cyclophilin A* was used as a housekeeping gene to normalize data. (B) Expression levels of microRNAs in the different confluence phases of HT29. *RNU44* and *U6* snRNA were used as internal controls. (C) Expression levels of proliferation markers *c-MYC* and *CCND1* together with differentiation markers *I-FABP* and *KLF4* in the different confluence phases of Caco2. *Cyclophilin A* was used as a housekeeping gene to normalize data. (D) Expression levels of microRNAs in the different confluence phases of Caco2. *RNU44* and *U6* snRNA were used as internal controls. Comparison of groups was performed using Kruskal Wallis test followed by Dunn’s post-test. Results are expressed as mean ± SEM of 3 independent experiments. Data from groups sharing the same lowercase letters were not significantly different, whereas data from groups with different case letters were significantly different (p<0.05).

The human HT29 and Caco2 colon carcinoma cell lines were used as a cellular system resembling the crypt-villus axis in terms of gene signature mirroring the fine balance between proliferation and differentiation. To validate the candidate miRNAs found in the array, we first selected the murine miRNAs showing human sequence homology using the database miRBase (www.mirbase.org/). The miRNAs *miR-7b*, *miR-199b-5p* and *miR-486* did not show any conserved sequence in humans, and for this reason they were left out. We focused our analysis on *let-7e*, *miR-378*, *miR-29b* and *miR-22*, which have showed high sequence homology. First, we assessed whether known genes involved in proliferation and differentiation were expressed according to the differentiation achieved in the HT29 and Caco2 system. As expected, the differentiation markers *I-FABP* and Kruppel-like factor 4 (*KLF4*) presented an increased expression along the pre-confluence, confluence and post-confluence phases, while the proliferation markers *c-MYC* and cyclin D1 (*CCND1*) displayed an opposite trend, with decreased expression along the above stated time points (Fig 3A–3C). After confirming the strength of the HT29 and Caco2 colon carcinoma cellular model, we proceeded with the validation of the miRNAs differentially expressed in our microarray on the intestinal fractions.

The miRNAs displaying increased expression going from the proliferative to the differentiated compartments (*miR-378*, *let-7e*, *miR-29b* and *miR-22*), showed an increased expression during the HT29 differentiation (Fig 3B). The expression of the selected miRNAs along the crypt-villus axis as well as in the HT29 differentiation model follow an opposite trend with respect to proliferative cell markers: while the latter diminish their expression during differentiation, miRNAs increase. To further corroborate the data observed in HT-29 cell line, we analyse the expression of the selected microRNA in Caco2 cell line, which retains high capability of differentiate *in vitro*. Accordingly, to that we observed for HT29, *let-7e* and *miR-29b* show a trend toward increase along with the differentiation process (Fig 3D). On the contrary, the level of *miR-22* and *miR-378* decrease during different phases of Caco2 differentiation. Taken together, our results point to a possible tumor suppressor role for *let-7e* and *miR-29b* in the intestine.

### 3.3 Expression of microRNAs in APC^Min/+^ mice

**Fig 4:**
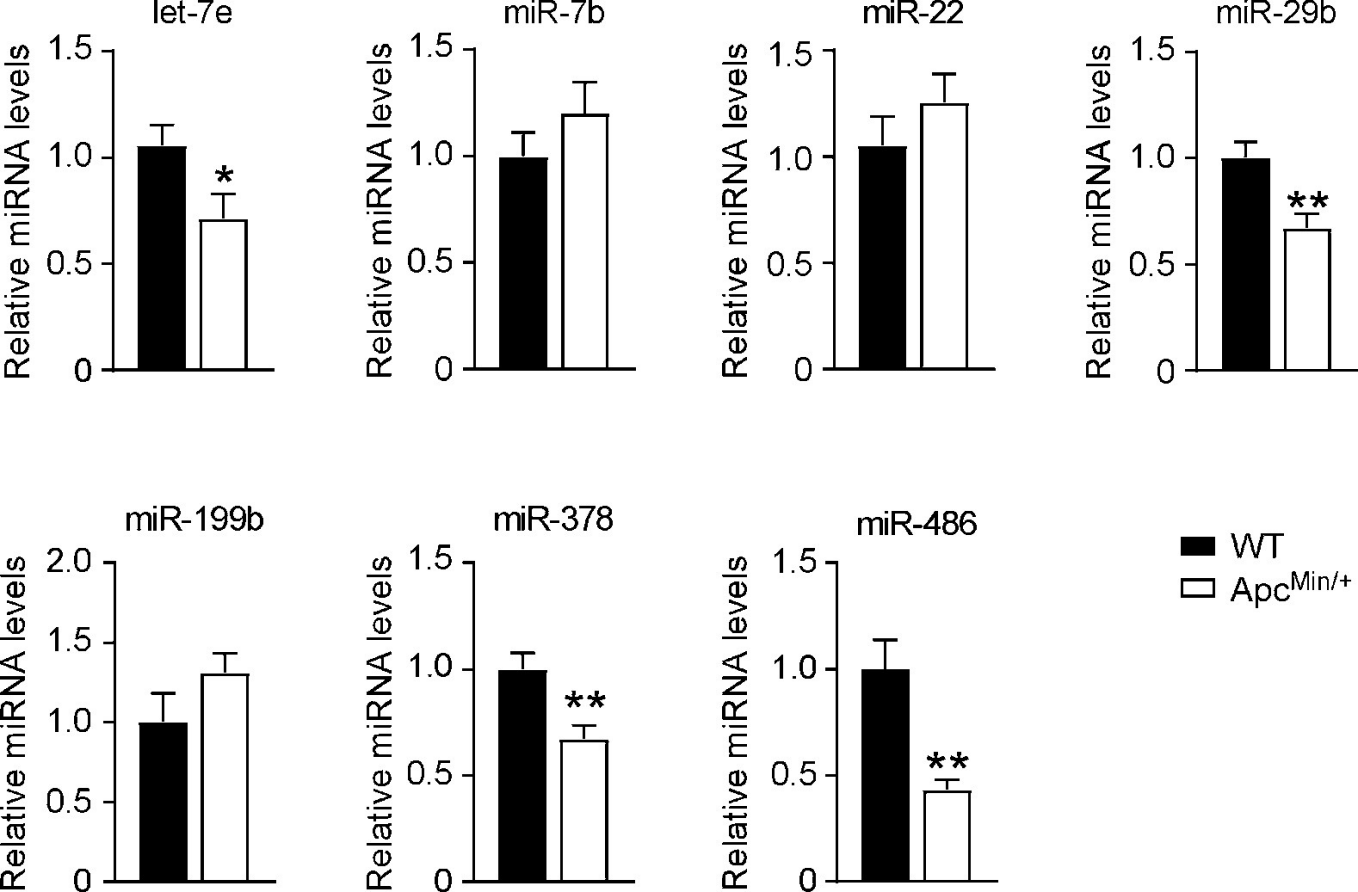
Expression of microRNAs in APC^Min/+^ mice. Ileum isolated from six months old APC^Min/+^ mice and age matched wild type controls was processed for RNA extraction. The expression levels of microRNAs were tested by Real Time qPCR. Quantitative normalization was performed using *sno202* as internal control. Comparison of different groups (n = 9) was performed using Mann-Whitney test. Results are expressed as mean ± SEM (*p<0.05).

Apc^Min^ is a point mutation in the murine homolog of the *APC* gene. APC^Min/+^ mice are a genetic model of colorectal cancer that develop spontaneous multiple intestinal adenomas, resembling humans carrying germ-line mutations in *APC* [22]. To test whether the identified miRNAs are involved in intestinal cancer suppression, we analysed their abundance in ileum tumor specimens harvested from APC^Min/+^ (9.18±2.86, tumors±SEM *per mouse*) and wild type control using Real Time qPCR (Fig 4). Intriguingly, *let-7e*, *miR-29b*, *miR-378* and *miR-486* are significatively downregulated in mouse model of cancer compared to WT mice, further corroborating the involvement of these microRNA in differentiation and cancer suppression programs. On the contrary, *miR-7b*, *miR-22* and *miR-199b* displayed a trend towards increase in this murine cancer model.

### 3.4 Expression of microRNAs in adenoma and colorectal carcinoma specimens

**Fig 5:**
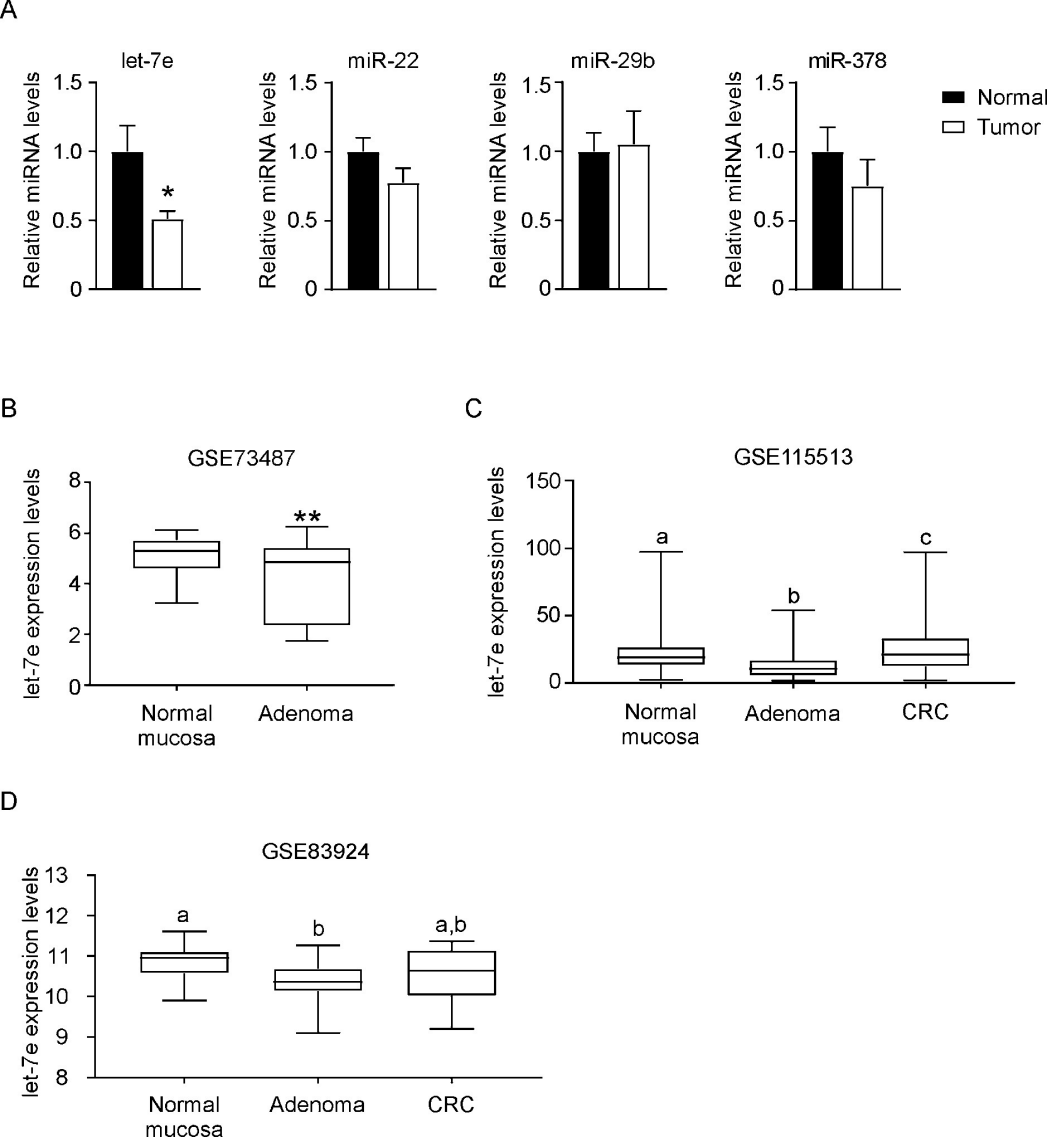
Expression of microRNAs in adenoma and colorectal specimens. (A) Real Time qPCR analysis of microRNA differentially expressed in polyp tissue compared to adjacent normal intestinal mucosa collected from FAP patients. Quantitative normalization was performed using *RNU44* and *U6* snRNA as internal controls. Comparison of different groups (n = 9) was performed using Mann-Whitney test. Results are expressed as mean ± SEM (*p<0.05). (B) *let-7e* expression in normal colon mucosa, adenoma and colorectal carcinoma quantified by microarray experiments. These data are extracted from deposited dataset published online (Accession Number: GSE73487; GSE83924; GSE115513). Data are displayed as box and whiskers, with median, minimum and maximum quartile.

To confirm the possible translational relevance of *let-7e*, *miR-29b*, *miR-22* and *miR-378*, which have been found differentially modulated along the crypt-villus axis and *in vitro* during post-confluence growth arrest, we evaluated by Real Time qPCR their mRNA abundance in polyp tissues (vs. normal adjacent mucosa) of 9 FAP patients. Except *miR-29b*, *miR-22*, *let-7e* and *miR-378* showed a strong trend towards down-regulation in the tumor tissue compared to the adjacent normal mucosa, with *let-7e* displaying a significative decrease expression, potentially supporting a tumor-suppressor role of these miRNAs (Fig 5A). Overall, these data show the involvement of these miRNAs, and in particular *let-7e*, in the modulation of intestinal differentiation/proliferation processes. To further corroborate our data on *let-7e*, we evaluate the abundance of *let-7e* in normal colonic mucosa, adenoma and colorectal carcinoma by analysing different deposited microRNA microarray (Accession Number: GSE73487; GSE83924; GSE115513). In all the examined dataset, the expression of *let-7e* was significantly lower in colonic adenoma than in normal specimens (Fig 5B). However, with the progression of tumor to overt colorectal carcinoma the level of *let-7e* increases, being closer to the one observed in normal tissue.

### 3.5 MicroRNA *let7-e* predicted target genes

**Fig 6:**
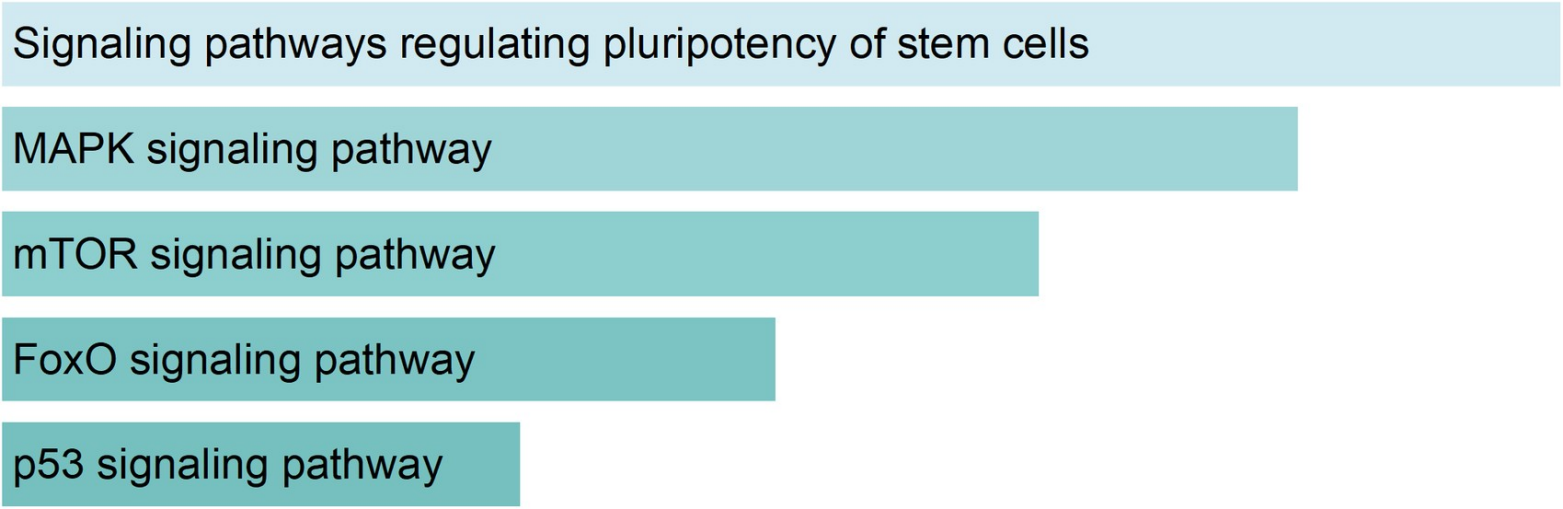
MicroRNA *let-7e* target prediction and enrichment analysis of the predicted target genes. Genes targeted by differentially expressed *let-7e* were predicted using TargetScan database. The gene set enrichment analysis was performed using EnrichR. The bar graphs display the principal predicted mouse KEGG pathways based on p-value ranking.

The results obtained both *in vitro* and *in vivo* point to a possible involvement of *let-7e* in differentiation processes, while lower *let-7e* levels are likely characteristic of early phase colonic adenoma in APC^Min/+^ mice and human FAP subjects. To further corroborate this hypothesis, we predicted the genes targeted by *let-7e* using TargetScan database. Then, we performed a gene set enrichment analysis using EnrichR. Interestingly, genes potentially modulated by *let-7e* are involved in the regulation of pluripotency in stem cells (Fig 6; S1 Table).

## 4. Discussion

The mammalian gut epithelium undergoes continuous and rapid cell renewal to guarantee the principal intestinal functions, such as absorption and defensive capacity, and finally tissue homeostasis. The entire series of cell renewal is paired to cell migration along the crypt-villus axis. Indeed, the stem cell population located at the basis of the crypt constantly divide to originate daughter cells that differentiate and migrate along the villus, and are finally extruded in the intestinal lumen after death [1].

Since today, several factors involved in numerous biological intestinal processes have been identified. These molecules contribute to the fine tuning of cell proliferation, cell differentiation, and cell death. Therefore, possible alterations concerning their expression and/or function may lead to the development of different diseases, including cancer. In the last decades, microRNAs have been identified as essential factors in the maintenance of intestinal homeostasis [20,23]. Indeed, not only miRNAs are differentially expressed between crypt and villus compartment, but also miRNA expression profiles are changed between undifferentiated and differentiated cell lines, thus indicating a possible role in determining the intestinal epithelial cell fate [21,24].

In the present study, we analysed microRNAs differentially expressed between crypt and villi compartment in mice ileum by microarray, and we validated the prominent ones, with a deep involvement in networks and pathways of cellular proliferation and/or differentiation, along the entire crypt-villus axis by Real Time qPCR. Our analysis considered only male mice, and not female one in order to limit the miRNA expression variability due to feminine hormonal cycle [25]. The microRNAs emerged from our analysis differs from those highlighted by Zhang and colleagues, probably due to the different methodology utilized in our studies [21]. With the aim to translate our results on human, we then employed an *in vitro* model resembling the crypt-villus axis, using HT29 and Caco2 colon carcinoma cell lines undergoing spontaneous differentiation due to postconfluence-induced growth arrest [26]. We analysed changes of genes (*c-MYC*, *I-FABP*, *CCND1* and *KLF4*) and selected miRNAs expression (*let-7e*, *miR-378*, *miR-22* and *miR-29b*) which showed a conserved sequence between mouse and human in preconfluence, confluence and postconfluence HT29 and Caco2 (Fig 3). Notably, let-7e and miR-29b show a trend toward increase across the various stages in both cell lines, thus suggesting an involvement in the differentiation process.

The intestinal stem cells residing at the bottom of the crypt play a crucial role in intestinal homeostasis, since they sustain the epithelial turnover along the crypt-villus axis, and their interplay with the environment impacts on the differentiation grade [27]. Moreover, intestinal stem cells can act as a cell of origin for intestinal tumorigenesis [28–31]. This process is characterized by an uncoupled mechanism of hyperproliferation to the detriment of differentiation. In human, colorectal cancer (CRC) represents the clinical manifestation of this imbalance. Usually occurring as adenomas that can eventually progress to malignant lesions called carcinomas. The majority of CRC occurs sporadically, while the remaining 30% arise as a consequence of inherited predisposition, such as the familial adenomatous polyposis (FAP). FAP patients develop hundreds of colon and rectum adenomas since young age, and CRC in untreated patients, due to a germline loss of function mutation in the APC gene [32].

To evaluate if microRNAs differentially expressed in the crypt-villus axis can be associated to tumorigenic lesions in the mice, we evaluated microRNAs expression in Apc^Min/+^, a murine model of cancer. These mice phenotypically reflect the intestinal manifestation observed in FAP patients, developing adenomas predominantly in the small intestine [33]. Three out of seven microRNAs (*miR-7b*, *miR-22* and *miR-199b*) display a slight trend to increase in ileum collected from APC^Min/+^ mice, while the others (*let-7e*, *miR-29b*, *miR-378* and *miR-486*) are significatively downregulated in cancerous lesions, thus indicating a closer association of these microRNAs to differentiation process rather than proliferation one (Fig 4).

Then, to translate our findings in human *in vivo*, we evaluated miRNAs expression in CRC samples from FAP patients. Notably, we found only *let-7e* significatively downregulated in tumor specimens, while the other miRNAs do not display any significative changes (Fig 5). Intriguingly, the analysis of deposited microarray revealed that the expression of *let-7e* is diminished in colon adenoma compared with normal mucosa. Conversely, CRC display higher *let-7e* level than adenoma.

The evaluation of the putative genes regulated by *let-7e* revealed an enrichment of those genes involved in the regulation of cells pluripotency, including Insulin-like Growth Factor 1 Receptor *(IGF1R)* and Activin A Receptor Type 1B *(ACVR1B)*. Remarkably, *IGF1R* has been found negatively regulated in *let-7e* overexpressing HCT-116 and LoVo cell lines [34,35]. Moreover, *let-7e* expression negatively correlates with *ACVR1B* in gastrointestinal stromal tumors (GIST) [36]. Altogether these results confirmed our observation in mice, and further sustain a possible involvement of *let-7e* in the differentiation process of intestinal epithelial cells rather than in the proliferation ones.

Intriguingly, other members of the conserved *let-7* microRNAs family have been so far characterized as regulators of cellular differentiation. Barely detected during embryogenesis, *let-7a*, *let-7b* and *let-7c* increase significantly as tissue differentiates forming adult rat kidney [37]. Moreover, *let-7b* plays a pivotal role in neural stem cell differentiation and its loss reverses the phenotype causing cells dedifferentiation [38]. At the same time, *let-7e* is involved in adipose derived stem cell differentiation toward epithelia [39]. Furthermore, downregulation of *let-7* family has been associated with metastases and poor prognosis in renal cell carcinoma as well as in glioblastoma, and *let-7e* deregulation in epithelial ovarian cancer cell lines promotes the development of resistance to cisplatin [40–42]. The increase expression of *let-7e* reverses the epithelial-to-mesenchymal transition in gentamycin-resistant pancreatic cell line [43]. Different studies have been performed to assay the role of *let-7e* in CRC. However, none of these studies took in consideration the early stage in transformation typical of adenomas, but rather focused on late stage tumors. The induction of *let-7e* suppresses HCT116 and HCT8 colon cancer cells proliferation and migration *in vitro* [35,44], while inhibition of *let-7e* resulted in increased cell growth [45]. Contrarily, the overexpression of *let-7e* in Caco2 cell line exerts an opposite effect, promoting metastases likely by targeting *E-cadherin* [46,47]. Whether these contradictory results are ascribable to the differentiation status of the cells used is not fully understood. In this view, our results depicting a unique physiological regulation of *let-7e* in different intestinal compartments as well as a downregulation of this miRNA in early phase colonic adenoma may help to shed a light into the effect of *let-7e* in tumor onset and progression.

Notably, high level of *let-7e* improves CRC radio- and chemosensitivity, and therefore limit tumour progression, via a direct interaction with IGF1R [34,35,46]. Intriguingly, sub-populations of chemoresistant cells in solid tumours present same biological peculiarities of cancer stem cells, responsible for tumor relapse and invasion [48].

Currently, it is believed that cancer stem cells originate from stem cell progenitors of the tissue of origin, and that tumour progression is characterized by a progressive differentiation of a cell which finally acquired stem-like properties [49]. In this view, *let-7e* may represent a useful diagnostic marker to characterize different stage of CRC, with higher expression in T1 stage of more differentiated cells than in T4 stage, where cells display mesenchymal and metastatic characteristic. Therefore, a downregulation of *let-7e* level may be used in the clinical practice to discriminate early stages adenoma, as emerged from the data displayed here.

## 5. Conclusions

Intestinal homeostasis consists of a fine balance of cell proliferation, cell differentiation and cell death. Intestinal stem cells residing at the bottom of the crypt are responsible for the entire epithelial renewal, occurring every 3–5 days. MicroRNAs are essential to maintain tissue homeostasis. MiRNAs more expressed in the crypt compartment are closely associated with proliferation, whereas those with high expression in the tip of the villi are involved in the differentiation and apoptosis processes. Intriguingly, alterations occurring to stem cells give rise to intestinal tumours, characterized by an imbalance between proliferation, differentiation and apoptosis programs.

In particular, here we identified *let-7e* as one of the principal miRNAs involved in differentiation process, since its expression increased along the crypt-villus axis and it predictively regulates genes involved in pluripotency of stem cells. Intriguingly, less *let-7e* characterizes early-stage colonic adenoma, such as the ones arising in APC^Min/+^ mice and FAP patients. Thus, assaying this miRNA could represent a rapid valuable option to discriminate tumor stage and eventually to distinguish early-stage adenoma.

## Supporting information

S1 Table Genes potentially regulated by *let-7e*. List of the *let-7e* putative target genes in the principal predicted mouse KEGG pathways based on *p-value* ranking. Genes targeted by differentially expressed *let-7e* were predicted using TargetScan database. The gene set enrichment analysis was performed by EnrichR.
(DOCX)
