# Supplementary material for: let-7e downregulation characterizes early phase colonic adenoma in APCMin/+ mice and human FAP subjects
Source: PLoS One. 2021 Apr 26;16(4):e0249238. doi: 10.1371/journal.pone.0249238 (PMC8075207; doi:10.1371/journal.pone.0249238)
Supplement: S1 Table — List of the let-7e putative target genes in the principal predicted mouse KEGG pathways based on p-value ranking. Genes targeted by differentially expressed let-7e were predicted using TargetScan database. The gene set enrichment analysis was performed by EnrichR. (DOCX) [file pone.0249238.s001.docx]

| **Pathway** | **P-value** | **Genes** |
| --- | --- | --- |
| Signaling pathways regulating pluripotency of stem cells | 5.79E-08 | ONECUT1; ACVR1B; IGF1R; NRAS; ACVR1C; AKT2; SMARCAD1; DVL3; WNT1; SKIL; SMAD2; APC2; FZD3; FZD4; PCGF3; STAT3; WNT9B; IGF1; WNT9A; DUSP9; ACVR2B; ACVR2A; MEIS1; PIK3CA; HAND1; HOXB1; BMPR1A |
| MAPK signaling pathway | 1.36E-06 | PDGFB; FASLG; NLK; CACNA1E; RASGRP1; DUSP16; IGF1R; ELK4; FGF5; RPS6KA3; PPP3CA; CACNA1I; NRAS; PAK1; MAPK8; AKT2; CASP3; MAP4K3; CACNG4; MAP4K4; DUSP4; MAP3K2; MAP3K3; MEF2C; MAP3K1; CHUK; DUSP1; INSR; IGF1; NGF; DUSP9; TGFBR1; DUSP7; CACNB4; TAOK1; FAS; TAB2; MAP3K13; TP53; CRK |
| mTOR signaling pathway | 4.41E-06 | PRKAA2; IGF1R; RPS6KA3; NRAS; AKT2; DVL3; SLC38A9; RICTOR; WNT1; FNIP1; RNF152; FNIP2; ATP6V1C1; FZD3; MIOS; CHUK; FZD4; INSR; WNT9B; TSC1; IGF1; WNT9A; PIK3CA; RRAGD; ULK2 |
| FoxO signaling pathway | 1.31E-05 | SMAD2; IL10; PRKAB2; CDKN1A; PRKAA2; CHUK; INSR; STAT3; IRS2; FASLG; IGF1; NLK; FBXO32; TGFBR1; IGF1R; NRAS; MAPK8; CCND2; CCND1; PIK3CA; AKT2; RAG1 |
| p53 signaling pathway | 2.10E-05 | STEAP3; CDKN1A; RRM2; IGF1; THBS1; CCND2; CDK6; CCND1; SESN3; CASP3; PMAIP1; FAS; MDM4; TP53; BCL2L1 |

**S1 Table. Genes potentially regulated by *let-7e.*** List of the *let-7e* putative target genes in the principal predicted mouse KEGG pathways based on *p-value* ranking. Genes targeted by differentially expressed *let-7e* were predicted using TargetScan database. The gene set enrichment analysis was performed by EnrichR.
